# S23. INTRODUCING COMPASS: COMPARING BRAIN ACTIVITY ACROSS PATIENTS WITH DIFFERENTIAL TREATMENT RESPONSE IN SCHIZOPHRENIA – AN OBSERVATIONAL STUDY

**DOI:** 10.1093/schbul/sby018.810

**Published:** 2018-04-01

**Authors:** Sandra Iglesias, Jakob Siemerkus, Martin Bischof, Sara Tomiello, Dario Schöbi, Lilian Weber, Jakob Heinzle, Julian Möller, Stephan Egger, Wolfgang Gerke, Markus Baumgartner, Wolfram Kawohl, Stefan Borgwardt, Stefan Kaiser, Helene Haker, Klaas Enno Stephan

**Affiliations:** 1 University of Zurich & Swiss Federal Institute of Technology (ETH Zurich); 2 University of Zurich; 3 ETH Zurich; 4 University of Basel; 6 Clienia Schlössli AG, Oetwil am See; 7 Psychiatrische Dienste Aargau AG; 8 Hôpitaux Universitaires de Genève

## Abstract

**Background:**

Present pharmacological treatment approaches in schizophrenia rest on “neuroleptic” drugs, all of which act as antagonists at dopamine D2/D3 receptors but additionally display major variability in their binding capacity to neurotransmitter receptors (Van Os & Kapur 2009). At present, the choice of any particular drug does not rest on any principled criteria: Once individual treatment has been started, therapeutic efficacy is monitored clinically, and a switch to a different drug is initiated when clear improvements remain absent after a few weeks. It is presently not possible to predict in advance which patients will respond well to a particular drug and who will experience little or no benefit (Case et al. 2011; Kapur et al. 2012).

For instance, clozapine and olanzapine are often prescribed after other antipsychotics have shown to be ineffective in patients with schizophrenia or related disorders due to their pronounced side-effects. Both drugs, clozapine and olanzapine, share certain pharmacodynamic properties with comparatively low affinity towards dopamine D2-receptors, but very high affinity towards muscarinic receptors – a unique constellation that distinguishes them from other common antipsychotics. Importantly, previous studies have shown that a subgroup of schizophrenia patients might particularly benefit from these properties (Raedler et al. 2003, Scarr et al. 2009).

Here, we present an ongoing observational study (COMPASS) which builds on these observations and addresses the question whether functional readouts of dopaminergic and muscarinic systems in individual patients could enable personalised treatment predictions. Guided by the dysconnection hypothesis of schizophrenia (Stephan et al., 2009), which postulates aberrant interactions between NMDA receptors and neuromodulators like dopamine/acetylcholine, the COMPASS study adopts a neuromodeling approach. The focus is on EEG/fMRI paradigms and computational models with empirically demonstrated sensitivity for altered function of NMDA, dopamine and muscarinic receptors, respectively.

**Methods:**

To detect even small effect sizes, the study aims to recruit N=120 patients with schizophrenia who begin treatment with, switch to, or augment medication with olanzapine or clozapine. If possible, a replication sample (an additional N=120) will be recruited, too. Patients will be examined +/- 96h relative to treatment onset. Data acquisition encompasses the following measurements: Clinical interview, EEG (working memory, reward learning under volatility, auditory MMN under volatility, “resting”-state), MRI (optional; fMRI during auditory MMN under volatility, “resting”-state, and structural imaging), blood samples (genetic and biochemical analyses). After 2 and 8 weeks a clinical follow-up is conducted.

**Results:**

The study is ongoing.

**Discussion:**

The EEG/fMRI data will be analysed by computational models that infer functional states of glutamatergic, dopaminergic, and cholinergic systems (for review, Stephan et al. 2015). Model parameter estimates will serve as features in machine learning analyses of treatment prediction (Brodersen et al. 2014).

If successful, this proof-of-concept study will lead to clinically useful tests for predicting the efficacy of clozapine/olanzapine prior to or during very early treatment. This could have a significant impact on clinical management as it would enable predicting, at an early stage, the therapeutic benefit for individual patients. Our neuromodeling approach to individual predictions may thus provide a principled basis for treatment decisions, help spare side-effects and enable informed switches in treatment strategy.

